# Semantic Ambiguity Resolution in Patients With Bipolar Disorder—An Event-Related Potential Study

**DOI:** 10.3389/fpsyg.2018.00270

**Published:** 2018-03-06

**Authors:** Hanna Schneegans, Klaus Hoenig, Martin Ruchsow, Manfred Spitzer, Bernhard J. Connemann, Markus Kiefer

**Affiliations:** ^1^Department of Psychiatry and Psychotherapy, Heinrich Heine University Düsseldorf, Düsseldorf, Germany; ^2^Department of Psychiatry and Psychotherapy III, Ulm University, Ulm, Germany; ^3^Department of Psychosomatic Medicine and Psychotherapy, Ulm University, Ulm, Germany; ^4^Department of Psychiatry, Clinic Christophsbad, Göppingen, Germany

**Keywords:** bipolar disorder, semantic ambiguity resolution, semantic inhibition, event-related potentials, language

## Abstract

Deficits in inhibitory function are assumed to underlie psychopathology in bipolar disorder (BD), especially in states of mania. A subdomain of inhibition, semantic inhibition (SI), referring to the suppression of irrelevant word meanings, may underlie formal thought disorder, such as flights of ideas. In the present study, we investigated SI in patients with BD during semantic ambiguity resolution using behavioral and event-related potential (ERP) measures. We presented 14 patients with BD with current manic, hypomanic, or mixed clinical states and 28 healthy controls sequentially with word triplets containing either a homonym (e.g., “organ”) or a comparable unambiguous word (e.g., “piano”). Participants were instructed to make a decision whether or not the target word was related to the meaning field of the first two words. The inappropriate homonym meaning had to be inhibited to correctly perform the target decision. In addition to reaction times (RT) and error rates (ER), the N400 ERP component to the target, an electrophysiological index of semantic processing, was analyzed as measure of the amount of SI that had taken place. Analyses of the behavioral data revealed that BD patients exhibited an overall worse performance in terms of RT and ER. In the ERP data, we found differences in N400 amplitude to ambiguous and unambiguous conditions over the right hemisphere in patients with BD depending on target congruence: In incongruent trials, N400 amplitude was smaller in ambiguous than in unambiguous words. In congruent trials, in contrast, N400 amplitude was larger in ambiguous than in unambiguous words. Such ERP differences between ambiguous and unambiguous words were absent in controls. We conclude that N400 amplitude differences in the ambiguous and unambiguous conditions of the BD group may reflect insufficient suppression of irrelevant homonym meanings in the right hemisphere. Disturbed SI processes might contribute to formal thought disorder in BD.

## Introduction

In patients suffering from bipolar disorder (BD), deficits in cognitive inhibition are among the most robustly documented cognitive deficits, especially during manic and mixed states (Strakowski et al., 2009; Sole et al., 2011). Cognitive inhibition can be defined as “the stopping or overriding of a mental process, in whole or in part, with or without intention” (MacLeod, 2007), and is thought to underlie several phenomena as diverse as stopping a prepotent motor response, the suppression of unwanted memories (Anderson et al., 2004), or interference control, as assessed by the Stroop task. Since inhibitory deficits have not only been found during the acute course of the illness, but also, albeit to a lesser degree, in remitted patients in euthymic states (Hidiroglu et al., 2015), and in healthy first-degree relatives of bipolar patients (Schulze et al., 2011; Hidiroglu et al., 2015), they have been considered a promising candidate for a bipolar endophenotype (Bora et al., 2009).

Cognitive inhibition deficits have been conceptualized as a basic cognitive dysfunction underlying manic psychopathology, giving rise to the more complex illness-defining behavioral features, such as impulsivity, distractibility, reduced ability to delay gratification, and overspending (Larson et al., 2005; Degabriele et al., 2011; Hajek et al., 2013). In congruency with this line of reasoning, brain regions that have been functionally linked to inhibitory processes, such as the ventrolateral prefrontal cortex (VLPF), the dorsolateral prefrontal cortex (DLPC), and the right inferior frontal gyrus (IFG), have been frequently found to exhibit structural and/or functional alterations in BD patients (Collette et al., 2001; Frangou, 2005; Chikazoe et al., 2007; Phillips and Swartz, 2014; Roberts et al., 2017).

Paradigms applied to investigate deficits in cognitive inhibition in BD patients include classic examples of motor-response inhibition such as the Stop-Signal Task or the Go/No-Go Task, as well as more cognitively complex tasks that comprise the suppression of distracting semantic content, such as the Stroop Task (Kravariti et al., 2009) or the Inhibition Condition of the Hayling Sentence Completion Task (HSCT). The latter requires a semantically *unrelated* sentence closure to be provided by the subjects, thus suppressing a highly primed semantically related response (Wang et al., 2013).

Such semantic inhibition (SI) of irrelevant conceptual meaning as investigated in the HSCT particularly warrants further attention, as deficits in the conceptual domain might especially be linked to formal thought disorder typical of BD, such as flights of ideas, racing thoughts, and loose or tangential thought. Homonyms, i.e., words with two distinct meanings, represent another feasible means for investigating SI phenomena (Faust and Gernsbacher, 1996). The current theory of homonym processing holds that both meanings of a given homonym (e.g., *organ* as a musical instrument and *organ* in the anatomical sense) encounter initial activation, with the degree of initial activation being a function of both homonym-meaning dominance (i.e., the relative preponderance of one meaning over the other in language use, leading to either balanced or more polar homonyms, where the latter comprise a dominant and subdominant meaning) and context (Twilley and Dixon, 2000; Swaab et al., 2003). Linguistic expressions in which the context favors the subdominant homonym meaning have been hypothesized to place especially high demands on semantic processes (Lee and Federmeier, 2009): In such instances, a high level of initial activation exists for both homonym meanings, which requires the establishment of an activation gradient between alternate meanings, in order to enable selection of the contextually fitting homonym meaning. These cognitive demands are expressed in additional time required for such processing (where context favors the subdominant homonym meaning), and has been referred to as *subordinate bias* (Kambe et al., 2001).

The generation of a sufficient activation gradient is thought to be achieved by means of SI (sometimes also referred to as suppression) (Gernsbacher and Faust, 1991; Faust and Gernsbacher, 1996; Shivde and Anderson, 2001; Hoenig and Scheef, 2009). However, in instances where context favors the dominant homonym meaning, shear surplus activation of the dominant homonym meaning might be sufficient for the selection of the appropriate homonym, possibly rendering inhibition of the subdominant homonym meaning unnecessary (Swaab et al., 2003).

**FIGURE 1:**
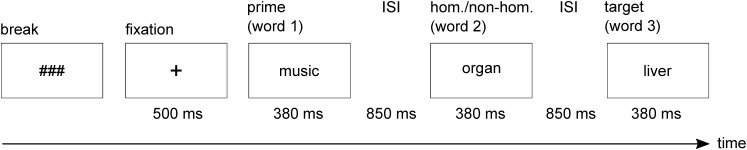
Example of a single trial used in the experiment (incongruent–ambiguous condition). hom., homonym; non-hom., non-homonym; ISI, interstimulus interval; ms, milliseconds.

In the present study, we investigated SI in bipolar patients in manic and mixed states and in matched healthy controls. We used a semantic decision paradigm requiring the selection of subdominant homonym meanings. This paradigm places high demands on SI, because for correct responding, participants must inhibit dominant homonym meanings. Participants were presented with word triplets consisting of a prime word, a homonym or an unambiguous word (word 2), and a target word, which was either congruent or incongruent with the meaning conveyed by the prime word and homonym/unambiguous word (**Figure 1**). In case of homonyms, the congruent target was always related to the subdominant meaning, whereas the incongruent target was always related to the irrelevant but dominant meaning of the homonym. Participants had to decide whether or not the target was related to the meaning field established by the prime word and the second word (homonym or unambiguous word). Hence, to correctly reject incongruent targets in the homonym condition as being unrelated, the irrelevant dominant meaning of the homonym had to be suppressed.

In addition to behavioral data (reaction time and response accuracy), event-related potentials (EPRs) were recorded to the targets to obtain an online measure of semantic processing. The centro-parietal N400 ERP component is a widely accepted index of semantic incongruity (Kutas and Hillyard, 1980; Kumar and Debruille, 2004). Its amplitude is inversely modulated by the degree to which a presented word semantically fits a given context: A greater mismatch between the meaning of a word and the semantic context is related to a larger N400 amplitude (Kutas and Federmeier, 2000).

N400 amplitude can also be taken to track previous inhibition of irrelevant word meaning as shown earlier (Gunter et al., 2003; Swaab et al., 2003; Lee and Federmeier, 2009): Within the context of the present experiment, N400 amplitude to the incongruent target word, which is semantically related to the inhibited dominant meaning of the homonym, should be a function of the residual activation of the dominant homonym meaning (Lee and Federmeier, 2009), and thus a quantitative index of the amount of SI that had taken place before. Small N400 amplitudes were to be expected if inhibition of the dominant homonym meaning was low, i.e., when the irrelevant meaning of the homonym is still activated to some extent. This would be indicative of an inhibitory deficit. Larger N400 amplitudes were to be expected when inhibition of the dominant, yet contextually inappropriate homonym meaning was more successful. Given the inhibition effects in patients with mania observed earlier, we expected in the patient group a smaller N400 amplitude to incongruent targets in the homonym condition than in healthy controls. For unambiguous words, N400 amplitude to incongruent targets should be comparable to control participants.

## Materials and Methods

### Participants

Fourteen patients (10 male) with a DSM-IV diagnosis of BD and current manic, hypomanic, or mixed psychopathology were enrolled in this study. Diagnoses according to DSM-IV comprised 296.4 (Bipolar I disorder, current or most recent episode hypomanic, *n* = 7), 296.43 (Bipolar I disorder, current or most recent episode manic, severe, without mention of psychotic behavior, *n* = 3), 269.44 (Bipolar I disorder, current or most recent episode manic, severe, specified as with psychotic behavior, *n* = 2), and 296.6 (Bipolar I disorder, current or most recent episode mixed, *n* = 2). The presence of an additional DSM-IV axis I disorder, besides a diagnosis of BD, as well as a diagnosis of substance abuse or dependence according to DSM-IV criteria, were exclusion criteria in the patient group. Most patients were inpatients from the Department of Psychiatry and Psychotherapy of the University Hospital Ulm and from the Clinic Christophsbad, Göppingen; one patient was an outpatient recruited with the help of a practicing psychiatrist via an advertising flyer.

For each patient, two healthy controls, matched for age, gender, and educational level (operationalized as years of schooling and higher education) were enrolled in the study as a control group (*n* = 28, 20 male), in order to increase statistical power. Control participants were free from any psychiatric disorder requiring treatment in the past as assessed by in-depth interviewing with an in-house questionnaire. Control subjects were recruited via newspaper advertisements and notice boards and received a small allowance for their participation (for further demographic data, see **Table 1**). Handedness of all participants was assessed using the German Version of the Edinburgh Handedness Inventory (Oldfield, 1971). In addition, verbal intelligence was measured with the WST (Schmidt and Metzler, 1992).

**Table 1:** Sociodemographic factors in participants; medication and clinical ratings in patients.

	Patients (BD)	Healthy controls (HC)
Gender (m/f)	10/4	20/8
Age, years (mean, *SD*, range)	39.6 (13.6, 20–65)	38.1 (13.5, 19–64)
Years of schooling (mean, range)	11.7 (1.7, 9–13)	11.3 (1.6, 9–13)
**Medication**		
Any antipsychotic agent (*n*%)	13 (92.9%)	
Chlorpromazine equivalents of AP (mean, *SD*, range)	338.1 mg (228.7, 0–800)	
Olanzapine (*n*%)	6 (42.9 %)	
Quetiapine (*n*%)	5 (35.7%)	
Risperidone (*n*%)	2 (14.3%)	
Perazine (*n*%)	2 (14.3%)
Haloperidol (*n*%)	1 (7.1%)
Any mood stabilizer (*n*%)	11 (78.6%)	
Lithium (*n*%)	7 (50.0%)	
Valproate (*n*%)	7 (50.0%)	
Any Benzodiazepine (*n*%)	5 (35.8%)	
Diazepam-equivalent doses (mean, *SD*, range)	10 mg (3.5, 0–15)	
YMRS (mean, *SD*, range)	16 (13.0, 3–36)	
BPRS (mean, *SD*, range)	43.9 (17.7, 22–73)	
BPRS item 4: formal thought disorder (mean, *SD*, range)	3.1 (2.1, 1–6)	
Duration of illness, years (mean, *SD*, range)	10.1 (8.0, 0–26)	
Age at first diagnosis, years (mean, *SD*, range)	29.6 (13.2, 16–61)	

*m, male; f, female; SD, standard deviation; AP, antipsychotic medication; YMRS, Young-Mania Rating Scale; BPRS, Brief Psychiatric Rating Scale.*

Both patient and control participants were native German speakers with normal or corrected-to-normal visual acuity. They were free from any neurological disorder with possible affection of the brain.

All patients with BD received psychotropic medication at the time of participation (details are presented in **Table 1**). Psychopathology in bipolar patients was assessed using the Young Mania Rating Scale (YMRS) (Young et al., 1978) and the Brief Psychiatric Rating Scale (BPRS) (Overall and Gorham, 1962).

Written informed consent was obtained from all subjects prior to participation. The study was conducted in accordance with the Declaration of Helsinki and was approved by the Ethics Committee of Ulm University. Data were collected in the context of a doctoral thesis by HS at Ulm University, Medical Faculty (Schneegans, 2015).

### Stimulus Material

Stimuli included word triplets consisting of a prime as word 1, a homonym or unambiguous word as word 2, and a target as word 3, which could be semantically congruent or incongruent to the meaning established by words 1 and 2. There were 160 triplets including homonyms as word 2, and 160 triplets including an unambiguous word as word 2. Altogether, stimuli included 80 prime words, 80 homonyms, 80 unambiguous words, and 160 targets. All 80 homonyms had been validated in a preceding pilot study (Wenke, 1998), in which a number of homonyms had been characterized for polarity (i.e., the degree to which one of the usually two distinct homonym meanings was more common in everyday language use than the other, allowing a distinction between balanced and polar homonyms) using a word-association task. The 80 homonyms chosen for this study were those that had been found to have a comparable high polarity. All homonyms were German nouns and both homographs and homophones.

Based on the 80 homonyms, four experimental conditions (ambiguous–incongruent; unambiguous–incongruent; ambi-guous–congruent; unambiguous–congruent; see also **Table 2**) were generated, with two of them providing no semantic fit (incongruent conditions) and two providing a semantic fit (congruent conditions) between the three words comprising the word triplet. Two of the four conditions contained a homonym as the middle word (ambiguous conditions), while the other two did not (unambiguous conditions). The same prime word was used in all four instances, and the target word was the same for both incongruent conditions (ambiguous, unambiguous) and for both congruent conditions (ambiguous, unambiguous). In the ambiguous–incongruent condition, target words were semantically related to the dominant homonym meaning, thus inducing a strong semantic interference and placing especially high demands on semantic processing.

**Table 2:** Conceptualization of the four experimental conditions.

Conditions		Word 1 (prime)	Word 2 (Homonym/unambiguous word)	Word 3 (target)
Incongruent	Ambiguous/Homonym	Music	Organ	Liver
	Unambiguous/Non-Homonym	Music	Piano	Liver
Congruent	Ambiguous/Homonym	Music	Organ	Choir
	Unambiguous/Non-Homonym	Music	Piano	Choir

Prior independent ratings of a group of subjects (*n* = 16) who did not participate in the actual experiment guaranteed equal semantic association between primes and the meaning of the second words, i.e., subordinate homonym meaning and meaning of unambiguous second word (Hellwig-Brida et al., 2007). Homonyms were matched to the unambiguous words with respect to word length and written word frequency (Baayen et al., 1993).

To avoid repetition effects, the total number of 320 triplets was divided into two sets of 160 triplets each, so that in one set each homonym, unambiguous word, and target word was presented only once to each participant. This could be achieved through combining the ambiguous–incongruent and the unambiguous–congruent condition of the first 40 homonyms with the unambiguous–incongruent and the ambiguous–congruent condition of the second 40 homonyms. Sets were assigned to participants in a balanced fashion so that each ambiguous and unambiguous word occurred with the same frequency in the congruent and incongruent conditions across participants. The use of two parallel material sets of 160 triplets each guaranteed that homonym polarity for congruent and incongruent ambiguous triplets was equal. Average relative frequencies of the subordinate homonym meanings (used for realization of either the congruent and incongruent triplets) were 26 and 27%, respectively. Finally, semantic association between target words and the field of meaning spanned by the preceding two nouns was equally strong for ambiguous and unambiguous triplets. Also, word length and word frequency of the three words were matched between ambiguous and unambiguous conditions across the two material sets.

### Experimental Design

Subjects were sitting in an electrically shielded and sound-attenuated booth. The 160 word triplets were presented in white font on a black background in the center of a CRT screen. Participants sequentially received the words of the triplets (**Figure 1**). Each word’s presentation duration was a function of its number of characters (14.7 ms per character) plus a constant of 300 ms. Average presentation duration of the words was 380 ms. Interstimulus interval between the words was 850 ms, during which the screen remained black.

After the appearance of the third word (target), participants were instructed to decide, as quickly and accurately as possible, whether the three nouns belonged to the same semantic field or not, by pressing one of two buttons labeled “yes” (belong to the same semantic field) or “no” (do not belong to the same semantic field). Participants then had to initiate the next trial via button press. Prior to the actual experiment, a practice session (24 trials not included in the main experiment, with immediate feedback) was administered to familiarize subjects with the task.

The trials were presented in two blocks, containing 80 trials each, which were separated by a short break. The break lasted up to 5 min, depending on individual participant’s needs. The order of the two blocks was counterbalanced across participants. Within each block, the trial order was randomized. Stimulus presentation and response recording was performed by a PC running a stimulation software package (Experimental Run Time System, BeriSoft Cooperation, Frankfurt, Germany) under MS-DOS.

### Acquisition of Behavioral Data and Analysis

Mean reaction times (RT) of correct responses and error rates (ER, relative frequency of errors) in each of the four conditions (ambiguous–congruent, unambiguous–congruent, ambiguous–incongruent, and unambiguous–incongruent) were calculated for the patient and the control groups, respectively. Errors were defined as wrong decisions, omissions, and decisions after a response deadline (>2.4 s after word triplet presentation).

Repeated-measures analyses of variance (ANOVAs) were performed, with the between-subject factor group (patients vs. healthy controls) and with the within-subject factors ambiguity (ambiguous vs. unambiguous word-triplets) and congruence (congruent vs. incongruent word-triplets).

Newman–Keuls tests were used for *post hoc* analyses, which control for false discovery rate related to multiple comparisons. Significance level of statistical testing was *p* < 0.05. For statistical analysis, Statistica (Version 12, StatSoft, Hamburg, Germany) was used.

### ERP Recording, Signal Extraction and Statistical Analysis

EEG was recorded from 64 Ag/AgCl electrodes, mounted in an elastic cap (Easy Cap, Herrsching, Germany), using BrainAmp amplifiers (Brain Products, Gilching, Germany) and BrainVision Recorder software (Version 1.03, Brain Products, Gilching, Germany). Electrodes were positioned with equal distances, so that some electrode positions do not correspond to the international 10–20 system, in which absolute distances between electrode sites are not equal. An electrode between Fpz and Fz was connected to the ground, and an electrode between Cz and FCz was used as recording reference. Eye movements were monitored with supra- and infra-orbital electrodes and with electrodes on the external canthi. Impedance on the individual electrodes was kept below 5 kΩ.

Data were continuously recorded at a sampling rate of 250 Hz and digitally filtered (0.01–70 Hz). Offline, EEG-data were further digitally filtered (low-cutoff: 0.1 Hz, 12 dB/oct and high cutoff: 30 Hz, 24 dB/oct). Independent component analyses (ICA) was used to remove ocular artifacts from the EEG (Makeig et al., 1996): In brief, we first transformed the EEG data into component space represented by 64 independent components. We then identified components reflecting blinks and vertical or horizontal eye movements according to their topography and their time course related to activity in the ocular channels. These ocular components were removed from the data, when transforming the data back from component space to conventional EEG space. Continuous data were segmented (with segments starting 150 ms prior and ending 1150 ms after word 3 presentation, target) and baseline-corrected (according to the 150 ms pre-stimulus interval). When we determined the epoch length for ERP analysis, we wanted to have the possibility to check whether later components than the N400 such as the late positive complex (LPC) would also be modulated by ambiguity. For that reason, we selected an epoch for EEG segmentation somewhat longer than necessary for analyzing the N400. Although it eventually turned out that only the N400 was modulated, as we initially expected, we decided to keep the longer epoch length.

Segments exhibiting amplitudes of more than 70 μV or less than -70 μV, respectively, were automatically excluded from further analysis as artifacts. Separately for each experimental condition, artifact-free EEG segments to trials with correct responses were averaged synchronous to the onset of the target. Thereafter, ERPs were re-referenced to average reference. The number of artifact-free epochs with correct responses, which were included into the averages, was generally high (range 33.4–38.5 out of 40), but significantly differed between groups and conditions as indicated by a repeated-measures ANOVA with the between-subject factor group (patients vs. controls) and the within-subject factors ambiguity (homonym vs. non-homonym) and congruence (congruent vs. incongruent). A main effect of “group”, *F*(1,40) = 7.46, *p* = 0.009, revealed a smaller mean number of analyzable segments in the patient (35.2) than in the control group (36.8). Furthermore, there were main effects of “congruence” *F*(1,40) = 5.73, *p* = 0.02 and “ambiguity” *F*(1,40) = 29.56, *p* < 0.001, which were due to a larger average number of analyzable segments in the incongruent (36.6) than in the congruent (35.3) condition as well as in the non-homonym (36.9) than in the homonym (35.1) condition. Other effects were not significant (*F* < 2.48, *p* > 0.13). These differences in the number of analyzable segments were mostly driven by differences in erroneous responses because they mirror the pattern found in the analysis of ER reported below.

For statistical analysis of the amplitude of the N400 ERP component, pairs of contralateral centro-parietal electrodes were selected (on the left hemisphere: CP1, CP3, C3; on the right hemisphere: CP2, CP4, C4), according to the known centro-parietal topography of the N400 (Kutas and Hillyard, 1980). In line with previous work, N400 amplitude was quantified as mean ERPs in a time window starting at 350 ms and ending at 500 ms after presentation of word 3 (target) and analyzed using repeated-measures analysis of variance (ANOVAs) with group (patients vs. controls) as between-subject-factor and hemisphere (left vs. right), electrode position (CP1/CP2, CP3/CP4, C3/C4), ambiguity (homonym vs. non-homonym), and congruence (congruent vs. incongruent) as within-subject factors. In analyses of behavioral and ERP data, violations of the sphericity assumption of the repeated-measures ANOVA were corrected using the Greenhouse-Geisser correction. If applicable, the original degrees of freedom were reported together with the Greenhouse-Geisser ε and the corrected *p*-value. Newman–Keuls tests were used for *post hoc* analyses. Significance level of statistical testing was *p* < 0.05. We also assessed how behavioral and ERP results were related to clinical variables in the patient group. To this end, the RT and ER differences between homonyms and non-homonyms in the congruent and incongruent conditions as well as corresponding N400 amplitude differences at left and right centro-parietal electrodes (averaged across electrode sites within each hemisphere) were correlated with duration of illness, severity of symptoms as measured by BPRS and YMRS as well as with chlorpromazine equivalents of antipsychotic medication using product-moment correlations.

## Results

### Clinical and Demographic Data

All patients received psychotropic medication at the time of participation in the study (**Table 1**), with the majority of patients receiving antipsychotics (*n* = 13) and mood stabilizers (*n* = 11). Some patients (*n* = 5) had received a benzodiazepine at the day of participation in the study. No intake of psychotropic drugs was registered in the control group.

Patients overall exhibited a moderate-to-low severity of manic and overall psychiatric symptoms, as reflected in a mean YMRS score of 16 and a mean BPRS score of 43.9 which in part was owed to the fact that patients needed to be sufficiently stable to comply with the experimental procedure, especially regarding EEG-recordings. The BPRS-item to measure formal thought disorder also showed a low mean score (3.1).

Patient and control groups did not differ significantly in terms of age [*t*(40) = 0.35; *p* = 0.73], years of school education [*t*(40) = 0.79, *p* = 0.44] and verbal intelligence, WST-score [*t*(40) = -1.43, *p* = 0.16]. Gender ratios were identical in the patient and control groups. With regard to handedness, two subjects in the patient group were left-handed in accordance with the Edinburgh Handedness Inventory, and one was found to be ambidextrous, while three control subjects were ambidextrous; all the remaining subjects were right-handed.

### Behavioral Data

**FIGURE 2:**
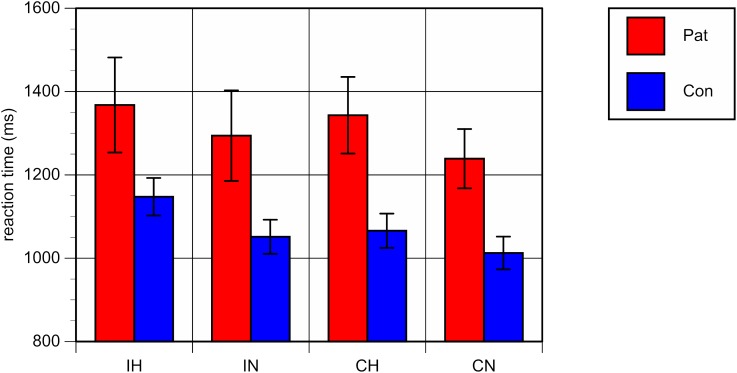
Mean reaction times (milliseconds) and standard errors for each of the four conditions. Patients with bipolar disorder in red, healthy controls in blue. IH, homonym incongruent; IN, non-homonym incongruent; CH, homonym congruent; CN, non-homonym congruent.

**FIGURE 3:**
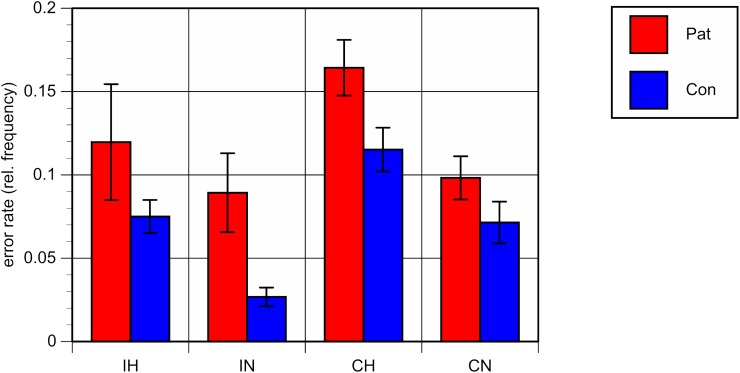
Mean error rates (errors per number of trials) and standard errors for each of the four conditions. Patients with bipolar disorder in red, healthy controls in blue. IH, homonym incongruent, IN, non-homonym incongruent; CH, homonym congruent; CN, non-homonym congruent.

Repeated-measures analyses of variance (ANOVA) with the between-subject factor “group” (patients vs. controls) and the within-subjects factors “ambiguity” and “congruence” on mean RT and ER showed a significant main effect for the factor “group,” with both higher RT [*F*(1,40) = 8.33, *p* < 0.01] and higher ER [*F*(1,40) = 10.53, *p* < 0.01] in patients as compared to healthy controls, reflecting an overall worse performance of patients in comparison with controls, irrespective of the specific conditions (see **Figures 2**, **3**).

A significant main effect was also found for the factor “ambiguity,” with both longer RT and a higher error rate in trials containing homonyms versus those that did not [RT: *F*(1,40) = 63.20, *p* < 0.001; ER: *F*(1,40) = 32.23, *p* < 0.001].

The interaction between “group” and “ambiguity” was not significant; for RT: *F*(1,40) = 0.47, *p* = 0.50; for ER: *F*(1,40) = 0.02, *p* = 0.89, indicating comparable behavioral costs of ambiguity processing in bipolar patients and in healthy controls. Regarding congruence, a main effect was only found with respect to ER, with more errors being made in congruent as compared to incongruent conditions [*F*(1,40) = 6.20, *p* < 0.05]. Correlation analyses of the RT and ER difference between homonyms and non-homonyms in the congruent and incongruent conditions in the patient group did not yield significant associations with clinical variables and chlorpromazine equivalents of antipsychotic medication. There was only a trend towards a correlation of duration of illness with the RT difference in the congruent condition (*r* = -52, *p* = 0.059).

### Electrophysiological Data, N400 Effects

**FIGURE 4:**
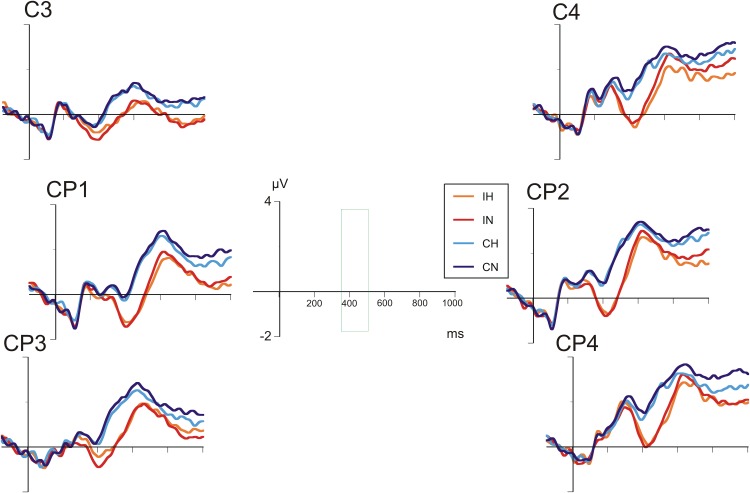
Grand average ERPs in healthy comparison participants at selected centro-parietal electrodes.

**FIGURE 5:**
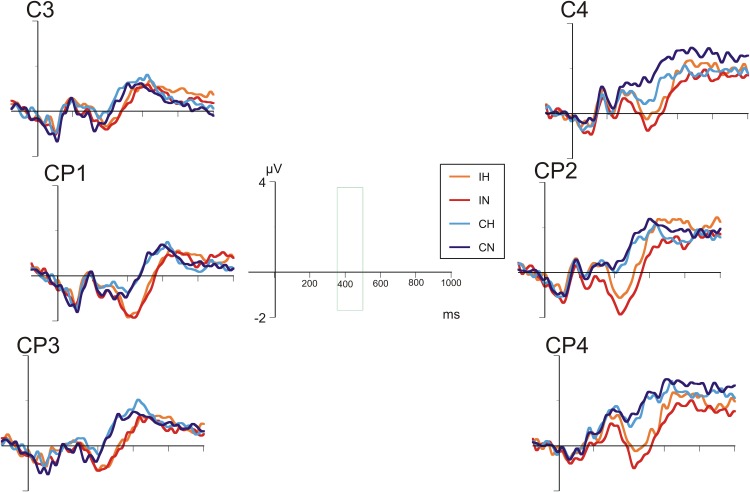
Grand average ERPs in patients with bipolar disorder at selected centro-parietal electrodes.

**Figures 4**, **5** show the grand average ERP waves in healthy controls and in patients with BD, respectively. On visual inspection, a greater negative amplitude can be seen for incongruent versus congruent conditions at all centro-parietal electrode positions in either group (N400 congruence effect). Only in patients with BD, over right centro-parietal electrodes (**Figure 5**), N400 amplitude for ambiguous versus unambiguous conditions differed depending on target congruency.

A repeated-measures analyses of variance (ANOVA) with the between-subjects factor “group” and the within-subjects factors “hemisphere,” “electrode position,” “ambiguity,” and “congruence” revealed a main effect for “congruence” [*F*(1,40) = 84.00, *p* < 0.001] indicating a more marked negativity in incongruent as compared to congruent conditions. In addition, there was a main effect for “hemisphere” [*F*(1,40) = 15.19, *p* < 0.001], with negativity overall being more pronounced over the left hemisphere.

Most importantly, the interaction between “hemisphere,” “ambiguity,” “congruence,” and “group” was significant [*F*(1,40) = 6.27, *p* < 0.001], reflecting ERP differences of ambiguous and unambiguous conditions over the right-hemisphere in patients with BD depending on congruence. *Post hoc* tests revealed significant differences between ERP waveforms of ambiguous and unambiguous conditions over the right hemisphere in patients with BD, both for the congruent (*p* = 0.011) and the incongruent (*p* = 0.023) condition. In the congruent condition, ERPs in the ambiguous condition were more negative than in the unambiguous condition. In the incongruent condition, however, ERPs in the ambiguous condition were less negative than in the unambiguous condition. Comparable differences between ambiguous and unambiguous conditions were absent over the left hemisphere in patients with BD and for healthy controls over either hemisphere. In both groups, incongruent targets elicited more negative N400 amplitudes than congruent targets in both ambiguous and unambiguous conditions (all *p*s < 0.001). Correlation analyses of the N400 ERP difference between homonyms and non-homonyms in the congruent and incongruent conditions at left and right centro-parietal electrodes in the patient group did not yield significant associations with clinical variables and chlorpromazine equivalents of antipsychotic medication, but correlations with BPRS (*r* = 0.53, *p* = 0.051) and YMRS scores (*r* = 0.52, *p* = 0.058) in the incongruent condition over the right hemisphere were close to statistical significance (all other *r*s < 0.40, *p*s > 0.16). Stronger symptom severity was associated with a reduced N400 amplitude (i.e., more positive ERP) for homonyms compared with non-homonyms indicating that the right cento-parietal N400 amplitude reduction found in the patient group in the ANOVA was more pronounced in patients with more severe psychiatric symptoms.

## Discussion

In our study, we assessed inhibition during semantic ambiguity resolution in patients with BD and matched comparison participants using ERP recordings. Participants were presented with a paradigm, which required them to inhibit the dominant meaning of polar homonyms in favor of the contextually relevant subdominant meaning in order to perform the correct semantic decision. As a control condition, participants performed an equivalent task with unambiguous words.

At a behavioral level, participants overall performed worse in ambiguous versus unambiguous conditions (with respect to both RT and ER). This suggests that SI processes needed to be employed for the selection of the context-adequate subdominant meaning of the homonym. Furthermore, patients with BD performed overall worse than control participants with respect to both RT and ER. This, however, is likely to reflect a general cognitive impairment, such as decreased processing speed (Vierck, 2015). In the present experiment, we did not detect a specific deficit in the processing of ambiguous trials in BD patients, as compared to HC. Thus, we could not find a selective impairment in SI at the behavioral level in BD patients, at least not in the moderately affected BD group examined here. This finding is in contrast to the meta-analysis of the Hayling Sentence Completion Test (inhibitory condition) by Wang et al. (2013), where SI deficits were reported. Task characteristics of the HSCT used in Wang’s study, however, differed considerably from ours. In the HSCT, word production as well as inhibition of a primed response was required, whereas in our paradigm the irrelevant dominant homonym meanings were to be inhibited to perform the correct semantic decision. Possibly, the inhibition demands are higher in the HSCT than in our paradigm and more strongly involve response inhibition. Unlike SI, response inhibition deficits have been quite frequently documented in patients with BD (Strakowski et al., 2009; Sole et al., 2011). It is also possible that the general impairment in performing our semantic decision task masked specific deficits in SI in the present paradigm at the behavioral level.

In the ERP data, there was a strong semantic congruence effect on N400 amplitude in both patients and controls: N400 amplitude was larger for incongruent than for congruent targets. This observation is in line with the known functional significance of the N400 component reflecting semantic activation and integration processes (Kutas and Federmeier, 2000). Most importantly, we found differences between patients with BD and healthy controls in the modulation of the N400 ERP component, which point to deficits of semantic ambiguity processing in the patient group. In the right centro-parietal electrodes, ERPs to ambiguous and unambiguous trials differed in patients with BD as a function of congruence. In the incongruent target condition, ERPs to ambiguous trials were less negative than to unambiguous trials. In the congruent target condition, ERPs to ambiguous trials were more negative in comparison to unambiguous trials. In control participants, ERPs to ambiguous and unambiguous trials did not differ. This N400 effect for ambiguous words in patients with BD suggests that in the patient group contextually inappropriate dominant homonym meanings are inhibited to a lesser extent and thus remain active in semantic memory to a higher degree. Interestingly, this right cento-parietal N400 effect to homonyms in the patient group tended to be correlated with psychiatric symptoms indicating that the SI deficit as observed in the ERP recordings is related to symptom severity. Although the localizational value of ERPs has to be viewed with caution, the present finding points toward a less restrictive mode of semantic processing in semantic areas of the right hemisphere.

In line with this interpretation, there has been evidence of differential contributions of each of the two hemispheres for language comprehension in general and the processing of homonyms in particular. It appears that the left-hemisphere plays the major role in inhibition of inappropriate homonym meanings (Burgess and Simpson, 1988; Faust and Gernsbacher, 1996), thus allowing for a more restrictive semantic processing, focusing activation on a single, most probable relevant sense (Meyer and Federmeier, 2007). Semantic processing in the right hemisphere is less restrictive, with broader activation patterns emerging in semantic memory, allowing, for example, priming for more distant semantically related words (Kiefer et al., 1998; Jung-Beeman, 2005). This major contribution of the left hemisphere is evidenced by priming experiments using divided visual fields, where semantic priming effects for both homonym meanings are found bilaterally at short SOAs, but in the right hemisphere only at longer SOAs. This suggests that SI of the irrelevant homonym meaning had taken place only in the left hemisphere, whereas in the right hemisphere both meanings were activated for a longer period of time (Burgess and Simpson, 1988; Faust and Gernsbacher, 1996). In line with this suggestion, Copland et al. (2002) found persisting semantic priming effects for contextually inappropriate homonym meanings in patients with lesions to the left hemisphere.

At the same time, there has been evidence for altered right-hemispheric function in BD patients (Najt and Hausmann, 2014). It can therefore be speculated that the degree to which right-hemispheric semantic processing contributes to overall semantic processing is increased in BD patients, with their general mode of processing resembling right-hemispherical language functions more closely. This could lead to a preponderance for more remote or indirect associations that might characterize some of the formal thought aspects seen in BD. The notion of increased activation of remote semantic associations has also been linked to increased creative output (Wu et al., 2016). Excessive right-hemisphere involvement in language comprehension has also been considered as an underlying feature in schizophrenic formal thought disorder (Faust and Kenett, 2014). Despite the common use of different psychopathological terms for their phenomenological descriptions, differentiation between schizophrenic and manic thought disorder based on specific linguistic properties has been proven difficult (Lott et al., 2002).

This study has several limitations: The sample size was small and the patients were inhomogeneous, especially with respect to exact diagnoses and handedness. However, it is unlikely that handedness affected the results because a differential lateralization of language functions depending on handedness would have influenced semantic processing of both homonyms and unambiguous words. In contrast, we found a specific right-lateralized N400 effect for homonyms. There were also differences in the number of analyzable artifact-free EEG segments with correct responses per participant group and conditions, which was mostly driven by differences in the number of erroneous responses, i.e., by a factor intrinsically associated with task performance. It is, however, unlikely that possible resulting difference in the signal-to-noise ratio of the ERPs compromised our findings, because the number of analyzable segments was generally high (33–38 out of 40 segments) and differences between critical conditions within and across groups were low (about 1–2 segments on average). Symptom severity at the time of examination was moderate, in part owing to the fact that EEG recordings have a low tolerance for excessive psychomotor activity, as seen frequently in more severely affected manic patients. Since symptom severity obviously is an important variable (Quraishi and Frangou, 2002), this could explain why we found deficits of semantic-ambiguity resolution in patients with BD only in the electrophysiological recordings, but not in behavioral performance. As a further caveat, it should be mentioned that patients received antipsychotics, mood stabilizers, and benzodiazepines, which potentially affect EEG activity. However, equivalent dose of antipsychotic medication did not significantly correlate with the homonym effect in the patient group. Analog correlation analyses for mood stabilizers and benzodiazepines were not possible due to the lack of equivalent doses for mood stabilizers and the low number of patients receiving benzodiazepines. Furthermore, it is difficult to explain how medication would have specifically altered EEG activity related to homonym processing.

In summary, our study points to altered SI processes during semantic ambiguity resolution in bipolar patients. Further studies could further elucidate the respective hemispheric contributions of the left and right hemispheres to SI in healthy participants and in patients with BD.

## Author Contributions

KH, MK, and HS conceived the study and developed the design. KH, HS, MR, and BC collected the data. HS and KH analyzed the data. HS wrote the first draft of the manuscript. All authors interpreted the data, revised the manuscript, and approved the final version.

## Conflict of Interest Statement

The authors declare that the research was conducted in the absence of any commercial or financial relationships that could be construed as a potential conflict of interest.
